# Efficacy of Qingpeng ointment (a Tibetan medicine) for acute gouty arthritis: a multi-center, randomized, double-blind, placebo-controlled trial

**DOI:** 10.1186/s12906-023-04328-7

**Published:** 2024-01-04

**Authors:** Ya-xi Shang, Shu-feng Wei, Ke-peng Yang, Yuan Liu, Su Wei, Xia Dong, Xin-chang Wang, Zhi-min Xie, Ru-lu Fang, Li-na Liang, Xiu-feng Li, Lei Xu, Mu-zhi Chen, Kai-xian Zhang, Ji-yong Huang, Le Wang, You-guo Yang, Hong-li Liao, Gui-e Xing, Yu-ping Zheng, Xiao-fen Li, Jing-lian Lin, Cheng-qian Shi, Yong-ping Zeng, Li-dan Mo, Fan Sun, Xiao-peng Li, Zhuo Zhang, Kai Chen, Zhao-chun He, Jian-ping Liu

**Affiliations:** 1 Centre for Evidence-Based Chinese Medicine, Beijing University of Chinese Medicine, 11 Bei San Huan Dong Lu, Chaoyang District, Beijing, 100029 China; 2 Affiliated Hospital of Shaanxi University of Chinese Medicine, Shaanxi University of Chinese Medicine, Xianyang, China; 3 Fangshan Hospital, Beijing University of Chinese Medicine, Beijing, China; 4 The Second Affiliated Hospital of Zhejiang Chinese Medical University, Hangzhou, China; 5 Liuzhou People’s Hospital, Liuzhou, Guangxi Zhuang Autonomous Region China; 6 The National Research Center in Complementary and Alternative Medicine (NAFKAM), Department of Community Medicine, Faculty of Health Science, UiT The Arctic University of Norway, Tromsø, Norway

**Keywords:** Tibetan medicine, Qingpeng ointment, Acute gouty arthritis, Randomized controlled trial, Integrative medicine, Pain

## Abstract

**Background:**

This study aims to assess the efficacy and safety of Qingpeng ointment (QPO), a Tibetan medicine for alleviating symptoms in individuals with acute gouty arthritis (AGA).

**Methods:**

This study was a randomized, double-blind, placebo-controlled trial that involved individuals with AGA whose joint pain, as measured on a visual analog scale (VAS) from 0 to 10, was equal to or greater than 3. The participants were randomly assigned to either the QPO or the placebo group and received their respective treatments twice daily for seven consecutive days. In case of intolerable pain, the participants were allowed to use diclofenac sodium sustained-release tablets as a rescue medicine. The primary outcomes measured were joint pain and swelling, while the secondary outcomes included joint mobility, redness, serum uric acid levels, C-reactive protein levels, and the amount of remaining rescue medicine. Any adverse events that occurred during the trial were also recorded.

**Results:**

A total of 203 cases were divided into two groups, with balanced baselines: 102 in the QPO group and 101 in the placebo group. For joint pain, differences between the groups were notable in the VAS scores [1.75 (0, 3.00) versus 2.00 (1.00, 3.50); *P* = 0.038], changes in VAS [5.00 (3.00, 6.00) versus 4.00 (2.00, 6.00); *P* = 0.036], and disappearance rate [26.47% compared to 15.84%; *P* = 0.046] after treatment. Concerning joint swelling, significant between-group differences were observed in the VAS scores [1.00 (0, 2.30) versus 2.00 (0.70, 3.00); *P* = 0.032] and disappearance rate [33.33% compared to 21.78%; *P* = 0.046] at treatment completion. The QPO group exhibited a statistically significant mobility improvement compared to the placebo group (*P* = 0.004). No significant differences were found in other secondary outcomes. Five patients, four from the QPO group and one from the other, encountered mild adverse events, primarily skin irritation. All of these cases were resolved after dosage reduction or discontinuation of the medication.

**Conclusions:**

Compared to the placebo, QPO exhibits positive effects on AGA by alleviating pain, reducing swelling, and enhancing joint mobility, without causing significant adverse effects.

**Trial Registration:**

ISRCTN34355813. Registered on 25/01/2021.

## Background

Gout is an inflammatory arthritis characterized by the deposition of monosodium urate crystals in soft tissues and joints. It is a common condition in adulthood [1, 2], with an incidence rate ranging from 1.1 to 3.9% [3–7]. The prevalence of gout is on the rise globally due to changes in dietary habits and lifestyle, leading to an increased disease burden [8]. Acute gouty arthritis (AGA) manifests as severe pain, swelling, redness, and limited mobility of joints, often resulting in physical disability and reduced quality of life [9].

Current treatment options for AGA, as recommended in guidelines, include colchicine, glucocorticoids, and nonsteroidal anti-inflammatory drugs (NSAIDs) [10–12]. However, the use of these drugs may be limited due to potential side effects such as nausea, diarrhea, stomach pain, gastrointestinal bleeding, renal damage, infections, and cardiovascular events [13–16]. Moreover, gout patients frequently have comorbidities that may further complicate the use of these medications [17]. For individuals unable to tolerate these adverse effects, alternative or complementary therapies with fewer side effects are desirable. A systematic review has shown that Chinese herbs have demonstrated some efficacy in improving joint function in gout patients [18].

Tibetan medicine, an ancient traditional healing system practiced for over 2000 years, is based on the cultural beliefs and practices of Tibetan people in China [19]. This system comprises a unique theoretical framework and utilizes various locally sourced medicines, some of which possess distinct therapeutic properties due to their unique growth environments [20]. One such Tibetan medicine is Qingpeng ointment (QPO), an externally used product that gained approval from the China National Medical Products Administration in 1997. QPO contains several herbal ingredients, including *Herba Oxytropis Falcatae* (Jidou), *Rhei Spiciforme Radix* (Yadahuang), *Radix Aconiti Flavi Et Penduli* (Tiebangchui), *Chebulae Fructus* (Hezi), *Terminaliae Belliricae Fructus* (Maohezi), *Phyllanthi Fructus* (Yuganzi), *Benzoinum* (Anxixiang), *Caulis Tinosporae* (Kuanjinteng), and artificial *Moschus* (Shexiang). These ingredients exhibit various therapeutic properties, which include anti-inflammatory effects in *Herba Oxytropis Falcatae*, *Rhei Spiciforme Randix*, *Caulis Tinosporae*, and *Radix Aconiti Flavi Et Penduli* [21, 22]; inflammation control in *Chebulae Fructus*, *Terminaliae Belliricae Fructus*, *Phyllanthi Fructus*, and *Benzoinum* [23–25]; analgesic effects in *Herba Oxytropis Falcatae*, *Rhei Spiciforme Randix*, and *Radix Aconiti Flavi Et Penduli* [21, 22]; and reduction of swelling in *Herba Oxytropis Falcatae* [21]. QPO’s active compounds primarily consist of flavonoids, anthraquinones, alkaloids, and polyphenols, conferring anti-inflammatory, anti-swelling, and analgesic properties [26]. Indications for applying QPO include joint pain and swelling associated with osteoarthritis, rheumatoid arthritis, rheumatic arthritis, or gout. Several previous clinical trials on AGA have suggested that adding QPO to NSAIDs can improve the effects of relieving joint pain and swelling, and enhancing joint mobility [27–32]. However, these studies have some methodological deficiencies, such as inadequate beforehand sample size estimation, small sample sizes in general, lack of reported appropriate methods for generating random sequences, and absence of blinding for doctors and patients.

To establish reliable evidence regarding the effectiveness and safety of QPO for AGA, it is crucial to conduct a well-designed randomized, double-blind trial with rigorous methodology. Hence, we undertook this multi-center, randomized, double-blind, placebo-controlled trial to assess the efficacy of QPO in alleviating joint pain, swelling, movement dysfunction, and skin redness, and to evaluate its safety for patients with AGA.

## Methods

### Study design and setting

This study was a multi-center, randomized, double-blind, placebo-controlled trial prospectively registered in the ISRCTN registry (ISRCTN34355813) on 25/01/2021. Participants were recruited from three clinical centers: Fangshan Hospital, Beijing University of Chinese Medicine; the Second Affiliated Hospital of Zhejiang Chinese Medical University; and Liuzhou People’s Hospital.

### Ethics

This study was conducted following the Declaration of Helsinki. The research protocol was approved by the ethics committees of Fangshan Hospital, Beijing University of Chinese Medicine (Reference No. FZY LK-2020–015), the Second Affiliated Hospital of Zhejiang Medical University (Reference No. 2020-Y-003-IH01), and Liuzhou People’s Hospital (Reference No. GCP2021–015–01).

### Study process

Participants were enrolled in the outpatient departments of the three clinical centers. After providing written informed consent, patients with an acute gout attack underwent checks to determine their eligibility. Those who met the criteria were randomly allocated to the treatment or control groups. Baseline measurements for treatment efficacy were collected. Patients in the treatment group were given QPO, while the control group received a placebo ointment. Both groups used their respective ointments for seven days. In addition, diclofenac sodium sustained-release tablets (DSSRTs) were provided to all patients as rescue medication for instances of intolerable joint pain.

During the treatment phase, patients were instructed to keep a daily diary. This diary was to record when they applied either the QPO or placebo ointment and took DSSRTs. After completing the 7-day treatment, patients returned to the hospital for a follow-up evaluation. At this visit, any remaining study medications were collected from the patients. Data were also gathered on the amount of remaining study drugs, any usage of combination medications, and any observed adverse events during the treatment period. Finally, outcomes related to efficacy were assessed and documented.

### Participants

Patients with acute gout flares were checked for eligibility criteria after signing written informed consent. Patients were eligible only if they were 18–65 years old and met the gout classification criteria released by the ACR (American College of Rheumatology)/EULAR (European League Against Rheumatism) [33]. They were required to have a VAS (visual analog scale, a 0–10 scale) score for joint pain of ≥ 3 and an acute gout flare, where the duration between the onset of the gout attack and their enrollment is ≤ 1 week.

Women who were pregnant or breastfeeding, and patients with other arthritis, advanced cardiovascular, cerebrovascular, hepatic, or renal conditions, dementia, or psychiatric disorders were not included. Patients were also not eligible if they were allergic to the study drugs, or had ulcers on the skin of the affected joint. Patients who had newly-added uric acid-lowering medications for any reason in the past week, or concurrently engaged in other clinical trials were excluded.

### Interventions

Participants in the treatment group were administered the QPO, while those in the control group were given a placebo ointment. Both the QPO and the placebo ointment, sourced from TIBET CHEEZHENG TIBETAN MEDICINE CO., LTD., were indistinguishable in terms of color, scent, texture, and packaging (20 g/aluminum tube). The active ingredients of QPO include *Herba Oxytropis Falcatae* (Jidou), *Rhei Spiciforme Randix* (Yadahuang), *Radix Aconiti Flavi Et Penduli* (Tiebangchui), *Chebulae Fructus* (Hezi), *Terminaliae Belliricae Fructus* (Maohezi), *Phyllanthi Fructus* (Yuganzi), *Benzoinum* (Anxixiang), *Caulis Tinosporae* (Kuanjinteng), and artificial *Moschus* (Shexiang). In contrast, the placebo ointment contained glycerin, liquid paraffin, methylparaben, and food coloring. Users were instructed to apply the ointment twice daily, ensuring it covered the affected joint area with 0.3–0.5 cm thickness. This ointment was then gently massaged into the skin until fully absorbed. The treatment process continued for seven days.

Participants from both groups were provided with DSSRTs (Beijing Novartis Pharmaceutical Co., Ltd, 75 mg/tablet, ten tablets/box) to act as rescue medication. Patients in both groups were advised to use the rescue medication exclusively when the Visual Analog Scale for Pain (VAS-pain) reached or surpassed 7 points. These scores were self-assessed by the patients. They were instructed not to use the rescue medication when the joint pain became tolerable, indicated by a VAS-pain score of less than 7 points. The suggested dosage was one tablet at a time, once daily, and not more than twice daily.

### Outcomes

#### Primary outcomes

1. Joint pain: This was assessed using the VAS, where scores of 0 and 10 represent no pain and intolerable pain, respectively. Patients recorded their scores at baseline, during the treatment, and at treatment completion.
2. Joint swelling:
  1. The severity of joint swelling was evaluated using the VAS. Here, scores of 0 and 10 correspond to no swelling and intolerable swelling, respectively. Patients recorded their scores at baseline, during the treatment, and at treatment completion.
  2. Vernier calipers (Ruineng NR0139) were employed to measure the thickness and width of the affected joints at baseline, during the treatment, and after the treatment concluded. For certain joints, due to their unique location, it was feasible to measure only one dimension, either width or thickness. As such, only one dimension was recorded for these joints. For instance, only the width was documented for knee and ankle joints, while only the thickness was gauged for the dorsum of the foot and hand. As for the remaining joints, both width and thickness were captured.

#### Secondary outcomes

1. Joint mobility: Assessed using a scale of 0–4 points. The scores are interpreted as follows: 0 indicates normal mobility; 1 denotes slight mobility restriction, but the ability to carry out regular activities remains; 2 signifies moderate mobility restriction with difficulties in regular activities but normal daily functions are feasible; 3 points to severe mobility restriction rendering daily tasks challenging, accompanied by significant pain during joint movement; and 4 represents immobile joints. Patients evaluated their mobility at baseline, during treatment, and after treatment completion.
2. Joint redness: Evaluated on a 0–3 points scale, where 0 means normal coloration; 1 is slightly reddened; 2 is markedly red; and 3 is deeply red. Patients provided these assessments at baseline, during the treatment phase, and after the treatment.
3. C-reactive protein (CRP): This was determined using blood samples taken at baseline and after the completion of treatment.
4. Serum uric acid (sUA): Assessed based on blood samples drawn at the starting point and upon treatment completion.
5. Remaining rescue medicine amount: The leftover quantity of rescue medicine is calculated as 10 minus the total number of DSSRTs consumed over the 7-day treatment period. The number of DSSRTs used during the treatment was documented at treatment completion.

#### Safety monitoring

Vital signs and general physical examinations were taken at the beginning and end of the treatment. Any adverse events encountered in the study were meticulously documented.

### Sample size

This study determined the sample size based on joint pain and swelling. In a previous study [27], 47% of the treatment group (who received both QPO and diclofenac sodium tablets) and 25% of the control group (administered only diclofenac sodium tablets) exhibited a notable reduction in joint pain (joint pain score, measured using a self-developed scale, decreased by six points). Another study [28] reported that the joint swelling score, measured on a 0–3 point scale, was found to be 0.45 ± 0.37 after administering QPO and etoricoxib tablets, compared to 0.87 ± 0.64 following the use of vaseline ointment and etoricoxib tablets. Based on a 5% type-I error rate (*α* = 0.05) and a 90% power (*β* = 0.1), factoring in an anticipated 20% dropout rate, the sample size calculated based on joint pain was 206, and for joint swelling, it was 68, with an equal distribution between the two groups. Given these calculations, our trial’s final sample size was established at 206, allocating 103 participants to each group.

### Randomization and blinding

Participants were randomly divided into two groups at a 1:1 ratio. An independent statistician produced the random sequence using SAS 9.2. To maintain allocation concealment, drug boxes, all appearing identical and numbered sequentially, were used. According to the random sequence, either the QPO or the placebo was placed in these numbered boxes. Every box contained three tubes of ointment and a box of DSSRTs. A designated staff member in each hospital was responsible for storing and managing the study drugs. Investigators enrolled participants. After enrollment, the numbered drug boxes were distributed to participants based on their enrollment sequence. In this study, clinicians and patients were unaware of the treatment allocations.

### Statistical analysis

Data were collected from case report forms. The Epidata software was used for data entry and management, while statistical analysis was conducted using SAS 9.2 software. The efficacy analysis adhered to the intention-to-treat (ITT) method, with a significance threshold set at 5%. Continuous data that fit a normal distribution were presented as “mean ± standard deviation (*SD*)” and analyzed using a t-test.In contrast, non-normally distributed continuous data were expressed as “median (*M*) and lower quartile (*Q*_25_), upper quartile (*Q*_75_) ”, and evaluated using the Wilcoxon rank sum test. Categorical data were articulated as “frequency and percentage” and analyzed using either Fisher’s exact test or the Chi-square test. The last-observation-carried-forward (LOCF) method was applied to address missing values.

### Quality control

Before initiating recruitment, all researchers underwent training to ensure a consistent and accurate understanding of the study protocol. The training included explaining the study’s progression, treatment guidelines, standard operating procedures for outcome measurement, and original data documentation. At the baseline visit, the investigator or research assistant clarified the proper methods for using the study drugs and assessing outcomes for each participant. Throughout the treatment phase, the investigator or assistant consistently contacted participants to remind them of the correct drug usage according to the protocol and to inquire about any potential adverse events. Throughout the research, clinical research associates were assigned to each study center to routinely oversee and ensure adherence to the specified protocol.

## Results

### Participants

**Fig. 1:**
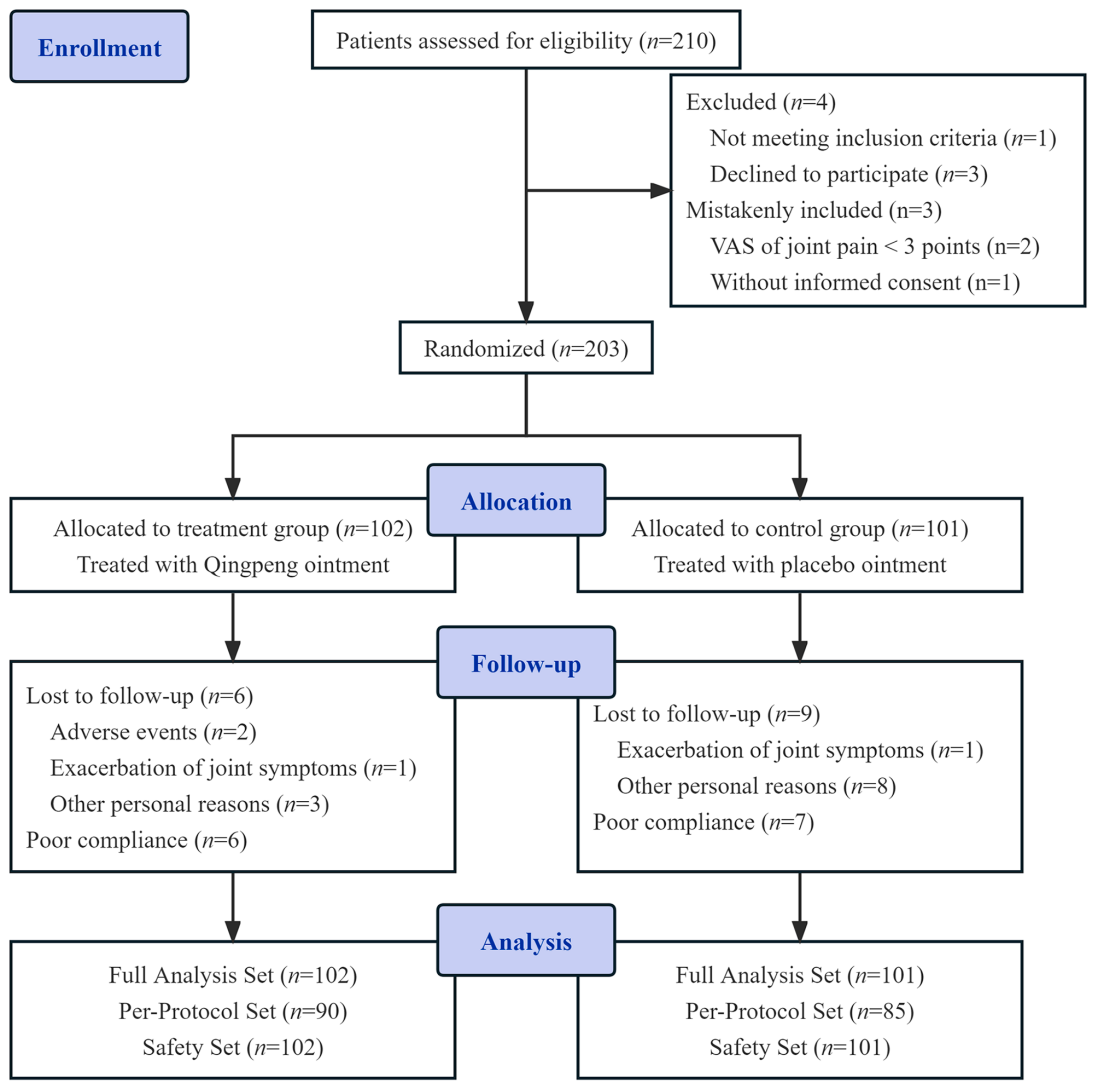
CONSORT flow diagram

The recruitment process of this trial started after registration, and finished in December 2021. A total of 210 participants were evaluated for eligibility. Out of these, four were excluded, and three were mistakenly included. The remaining 203 participants were randomly assigned to the QPO (*n* = 102) and placebo groups (*n* = 101). During the study, two participants withdrew due to the worsening of joint symptoms, two patients dropped out due to adverse events, and 11 participants dropped out due to other personal circumstances (such as demanding work schedules and business trips, family issues, COVID-19-related quarantines, or an unwillingness to maintain contact for unspecified reasons). Thirteen participants demonstrated poor compliance (Fig. 1). Ultimately, both the full analysis set (FAS) and safety set (SS) comprised 203 participants, while the per-protocol set (PPS) included 175 participants.

### Baseline characteristics

The demographic and disease characteristics, along with the study variables at the baseline, were well-balanced between the two groups. To assess the extent of swelling, joints were categorized by their positions, and subsequently, the swelling was measured for each joint type. Additionally, this analysis considered various other joints, such as those in the fingers, toes, wrists, and elbows. Table 1 presents the baseline characteristics of patients in both groups.

**Table 1:** Baseline characteristics of patients with acute gouty arthritis

	QPO group (*n* = 102)	Placebo group (*n* = 101)
Age (years)	41.00 (33.25, 50.00)	41.00 (32.00, 54.00)
Gender (Male/Female)	96 (94.12%)/6 (5.88%)	94 (93.07%)/7 (6.93%)
BMI (kg/m^2^)	26.21 (23.71, 29.03)	25.95 (24.22, 28.73)
Participants who have ever had gout	80 (78.43%)	78 (77.23%)
History of gout (years)	2.00 (0.35, 4.75)	2.00 (0.50, 4.00)
Time from gout attack to enrollment (days) ^a^	2.0 (1.0, 3.0)	2.5 (1.0, 4.0)
Participants who used other drugs before enrollment	28 (27.45%)	25 (24.75%)
Participants on urate-lowering therapy at baseline	7 (6.86%)	5 (4.95%)
VAS-pain	6.5 (5.0, 7.0)	6.3 (5.0, 7.3)
VAS-swelling	6.0 (4.0, 6.0)	6.0 (4.0, 7.0)
Joint position		
MP joints ^b^	50 (49.50%)	42 (42.00%)
Dorsum of the foot/hand	10 (9.90%)	10 (10.00%)
Knee/ankle joints ^c^	33 (32.67%)	39 (39.00%)
Other joints	8 (7.92%)	9 (9.00%)
Measure of swelling (mm)		
MP joints - width	92.20 (83.58, 96.50)	93.95 (86.63, 98.55)
MP joints - thickness	44.70 (37.98, 49.68)	43.65 (40.43, 49.93)
Dorsum of the foot/hand - thickness	45.82 ± 14.75	56.30 ± 16.90
Knee/ankle joints - width	79.74 ± 15.60	81.83 ± 15.30
Other joints - width	55.58 ± 27.55	53.78 ± 33.13
Other joints - thickness	43.76 ± 22.75	40.58 ± 26.21
Score of mobility	2.0 (1.0, 2.0)	2.0 (1.0, 2.0)
Score of redness	2.0 (1.0, 2.0)	1.0 (1.0, 2.0)
CRP (mg/L) ^d^	9.57 (3.64, 20.87)	5.50 (2.28, 19.86)
sUA (mmol/L) ^e^	481.0 (397.6, 573.0)	496.0 (379.0, 551.0)
Participants with sUA within normal range at baseline	23 (22.55%)	30 (29.70%)

QPO: Qingpeng ointment; VAS: visual analog scale; MP: Metatarsophalangeal; CRP: C-reactive protein; sUA: serum uric acid

^a^ Date of gout attack was not correctly recorded for four patients in the QPO group and one patient in the placebo group, and the time from gout attack to enrollment was not available for these patients

^b^ Baseline swelling measure of the metatarsophalangeal joint was not recorded for one patient in the QPO group

^c^ Baseline swelling measure of the ankle joint was not recorded for one patient in the placebo group

^d^ Baseline C-reactive protein was not recorded for seven patients in the QPO group and seven patients in the placebo group

^e^ Baseline serum uric acid was not recorded for one patient in the QPO group

### Efficacy

#### Pain

After the 7-day treatment, patients receiving QPO showed a significant reduction in VAS-pain compared to those receiving the placebo (1.75 (0, 3.00) in the QPO group, 2.00 (1.00, 3.50) in the placebo group, *P* = 0.038). Moreover, the change in VAS-pain from baseline to post-treatment was significantly higher in the QPO group (5.00 (3.00, 6.00) in the QPO group, 4.00 (2.00, 6.00) in the placebo group, *P* = 0.036). The disappearance rate of joint pain, indicated by the percentage of patients with VAS scores reduced to 0 out of the total number of patients, was notably higher in the QPO group (26.47%) compared to the placebo group (15.84%), with a *p*-value of 0.046.

#### Swelling

Regarding joint swelling, VAS-swelling at treatment completion showed a significant difference between the two groups, favoring the QPO group (1.00 (0, 2.30) in the QPO group, 2.00 (0.70, 3.00) in the placebo group, *P* = 0.032). However, the changes in VAS-swelling from baseline to treatment completion did not exhibit any significant difference between the two groups (*P* = 0.201). This lack of significance might be attributed to the very mild degree of joint swelling noted in some patients at baseline, and be affected by the patients’ observation through eyes. Similar to joint pain, the disappearance rate of swelling in the QPO group (33.33%) markedly increased compared to the placebo group (21.78%), with a *p*-value of 0.046. The results of VAS-pain and VAS-swelling are provided in Table 2.

**Table 2:** VAS (pain and swelling) at treatment completion and changes from baseline to treatment completion in two groups

	Treatment completion			Changes from baseline to treatment completion		
	QPO group (*n* = 102)	Placebo group (*n* = 101)	*P*	QPO group (*n* = 102)	Placebo group (*n* = 101)	*P*
VAS of pain	1.75 (0, 3.00)	2.00 (1.00, 3.50)	0.038	5.00 (3.00, 6.00)	4.00 (2.00, 6.00)	0.036
VAS of swelling	1.00 (0, 2.30)	2.00 (0.70, 3.00)	0.032	3.85 (2.00, 6.00)	3.00 (2.00, 5.00)	0.201

QPO: Qingpeng ointment; VAS: Visual Analog Scale

We analyzed the changes in swelling measures from baseline to treatment completion for various types of joints. Specifically, the results for metatarsophalangeal joints, dorsum of the foot and hand, knee and ankle joints showed no significant difference between the two groups. This observation may be attributed to the classification of participants based on the position of their affected joints, which resulted in relatively small sample sizes for each joint category (less than half of the total sample size). The detailed results of swelling measures are presented in Table 3.

**Table 3:** Changes in swelling measures from baseline to treatment completion in two groups

		QPO group	Placebo group	*P*
MP joints – width (mm)	(*n* = 50, *n =* 42)	1.40 (0.50, 4.00)	2.50 (0.20, 4.83)	0.340
MP joints – thickness (mm)	(*n* = 50, *n* = 42)	3.05 (1.00, 6.58)	2.60 (0.03, 5.10)	0.150
Dorsum of the foot/hand – thickness (mm)	(*n* = 10, *n* = 10)	2.05 (1.45, 10.83)	3.85 (2.00, 4.80)	0.485
Knee/ankle – width (mm)	(*n* = 33, *n* = 39)	2.60 (0, 5.10)	2.70 (0.10, 6.50)	0.496
Other joints – width (mm)	(*n* = 8, *n* = 9)	4.10 (2.85, 8.35)	1.60 (0.30, 3.50)	0.014
Other joints – thickness (mm)	(*n* = 8, *n* = 9)	5.65 (4.48, 8.25)	0.80 (0.40, 2.00)	0.003

CPO: Qingpeng ointment; MP: Metatarsophalangeal

#### Secondary outcomes

Following the 7-day treatment, the QPO group demonstrated a significantly greater decrease in mobility score compared to the placebo group (1.0 (1.0, 2.0) in the QPO group, 1.0 (0, 1.0) in the placebo group, *P* = 0.004). However, changes in redness scores (*P* = 0.952), CRP (*P* = 0.067), and UA (*P* = 0.533) from baseline to treatment completion did not show significant differences between the two groups. Likewise, the between-group differences in the remaining amount of rescue medicine did not reach statistical significance (*P* = 0.133). The results of the mobility score, redness score, CRP, UA, and the remaining amount of rescue medicine can be found in Table 4.

**Table 4:** Changes between baseline and treatment completion in the score of mobility, the score of redness, CRP, UA, and the remaining amount of rescue medicine in two groups

		QPO group	Placebo group	*P*
Score of mobility	(*n* = 102, *n* = 101)	1.0 (1.0, 2.0)	1.0 (0, 1.0)	0.004
Score of redness	(*n* = 102, *n* = 101)	1.0 (0, 1.0)	1.0 (0, 1.0)	0.952
CRP (mg/L)	(*n* = 95, *n* = 94)	2.52 (0, 12.25)	0.07 (0, 9.03)	0.067
UA (mmol/L)	(*n* = 101, *n* = 101)	-0.50 (–64.40, 42.00)	0 (–58.50, 19.00)	0.533
Remaining amount of rescue medicine (tablets)	(*n* = 102, *n* = 101)	8.5 (5.0, 10.0)	10.0 (5.0, 10.0)	0.133

QPO: Qingpeng ointment; CRP: C-reactive protein; UA: uric acid

### Adverse events

In the QPO group, four patients (3.92%) encountered adverse events, which included one case each of skin itching, rash with itching, skin redness and swelling, and one patient experienced dizziness, nausea, and palpitation. Meanwhile, the placebo group had one patient (0.99%) who experienced skin itching. However, the two groups had no significant difference in the occurrence of adverse events(*P* = 0.369). All these adverse events were mild and resolved either by reducing the dosage or discontinuing the use of the ointment.

## Discussion

### Summary of findings

Concerning joint pain, Tibetan medicine QPO demonstrated significant benefits compared to the placebo ointment in terms of VAS and symptom disappearance rate at the end of the treatment, as well as the change in VAS from baseline to the end of treatment. Moreover, for joint swelling, the VAS at treatment completion was notably lower, and the disappearance rate of swelling was markedly higher in the QPO group than in the placebo group. Among the secondary outcomes, the QPO group exhibited a more significant decrease only in the mobility score, while no notable differences were found regarding changes in the redness score, CRP, UA, and the remaining amount of rescue medicine. Regarding safety, four cases from the QPO group and one from the placebo group reported adverse events. These events manifested as mild skin irritation symptoms, dizziness, nausea, and palpitations. All the adverse events were effectively treated through dose reduction or discontinuation of the medication.

### Comparison with previous studies

Previous clinical studies have assessed the effectiveness of various topical Tibetan medicines, such as Xiaotong plaster, Xueshan Jinluohan analgesic coating agent, and Wuwei Ganlu medicated bath, for alleviating symptoms of AGA [34–36]. These studies demonstrated that incorporating topical Tibetan medicines alongside the first-line treatments recommended in guidelines can improve efficacy in relieving joint pain, swelling, and dysfunction.

In the context of AGA treatment, six clinical trials have examined the efficacy of QPO [27–32]. Among them, three studies found that combining QPO with NSAIDs enhanced effectiveness in reducing joint swelling, while one study reported that adding QPO to NSAIDs was more effective in alleviating joint pain. However, these trials focused solely on the combined use of QPO with NSAIDs and did not investigate the efficacy of QPO alone. Additionally, these previous studies exhibited methodological deficiencies, including improper random sequence generation, lack of blinding, and absence of pre-defined sample size estimation (varying from 50 to 95). This led to underpowered trials. Furthermore, the results and conclusions of these studies were inconsistent.

In contrast to previous research, the current study employed a randomized, double-blind, placebo-controlled trial design. This approach ensured that doctors and participants were unaware of the assigned treatment, and the sample size was predetermined based on prior research. Consequently, the efficacy of QPO for AGA was evaluated more rigorously. Another distinction lies in the intervention used in this study, which solely involved the application of topical ointment. To improve participant adherence, both groups were provided with DSSRTs as a rescue medicine. The amount of remaining rescue medicine after treatment served as a secondary outcome to assess whether QPO effectively alleviated joint pain. Lastly, joint swelling was evaluated using both the VAS and swelling measures (width and thickness of affected joints measured with vernier calipers). This innovative approach allowed for the subjective and objective measurement of joint swelling, setting this study apart from prior investigations.

### Implications for clinical practice

The use of first-line oral medications to treat AGA is limited due to their potential side effects [13–16]. In contrast, topical drugs act locally on the skin surface [37], providing a more direct relief of joint symptoms. Moreover, compared to oral drugs, topical medications may offer a safer alternative, as they have lower systemic absorption [37]. The main adverse reactions associated with topical drugs are mild to moderate skin irritation reactions at the application site [38], such as itching and rash. Prior studies have demonstrated effective acute pain relief with certain topical drugs [39], and topical NSAIDs have shown comparable efficacy to their oral counterparts [37]. Therefore, topical drugs present a suitable option for patients who cannot tolerate the adverse effects of some first-line oral medications. Combining oral drugs with topical therapies may also improve efficacy in managing AGA symptoms.

### Strengths and limitations

This study is the first multi-center, randomized, double-blind, placebo-controlled trial that assesses the effectiveness of QPO for AGA. Different from previous clinical trials, this trial offers more accurate and reliable evidence on the efficacy and safety of QPO for AGA, owing to a more rigorous design with a predetermined sample size, pre-registered protocol, and blinding of patients and doctors.

However, we acknowledge some limitations in our study. Regarding study design, firstly, the intervention and control in this trial involved only QPO and placebo ointment. However, AGA patients often experience acute and severe joint pain, and it might be challenging for them to rely solely on ointment for pain relief. Although oral drug DSSRTs were provided as rescue medicine, participant compliance might also be affected. Secondly, most outcomes in this trial were measured and reported by the patients. While investigators and research assistants offered detailed instructions for measuring these outcomes, it is still possible that one or two participants did not wholly comprehend the methods. This lack of understanding could lead to inaccuracies in outcome assessment. In the aspect of study implementation, patient compliance was not as expected. The analysis of drug application indicated that some patients did not adhere to the prescribed regimen. Specifically, five patients in the QPO group and four in the placebo group applied the ointment fewer than ten times during the treatment period, which should be applied 14 times. Lack of patient compliance might affect the results of efficacy assessment. Additionally, 15 patients withdrew from the study for various reasons; among them, two dropped out for adverse events, two for worsening joint symptoms, two for COVID-19-related quarantines, one for family issues, and one for a business trip. Seven patients chose not to maintain contact for unspecified reasons. Dropout of participants resulted in missing trial data. Although the LOCF method was used to handle missing values, the efficacy analysis could still be subject to biases.

## Conclusions

This double-blind, placebo-controlled trial found that QPO exhibits beneficial effects in relieving joint pain and swelling, and improving joint mobility, as compared to the placebo. The results suggest that QPO could serve as a safe treatment option for alleviating joint pain, swelling, and dysfunction in patients with AGA.

## Data Availability

The datasets used and analyzed during the current study are available from the corresponding author upon reasonable request.

## Abbreviations

ACR: American College of Rheumatology
AGA: Acute gouty arthritis
CRP: C-reactive protein
DSSRT: Diclofenac sodium sustained-release tablet
EULAR: European League Against Rheumatism
FAS: Full analysis set
ITT: Intention-to-treat
LOCF: Last-observation-carried-forward
MP: Metatarsophalangeal
NSAID: Nonsteroidal anti-inflammatory drug
PPS: Per-protocol set
QPO: Qingpeng ointment
SD: Standard deviation
SS: Safety set
UA: Uric acid
VAS: Visual analog scale
